# Triphenyl(3,4,5-trimethoxy­benzyl)­phospho­nium chloride monohydrate

**DOI:** 10.1107/S1600536808027712

**Published:** 2008-09-06

**Authors:** Alice M. Barkell, Jonathan Sharp, Stephen J. Simpson

**Affiliations:** a School of Biosciences, Geoffrey Pope Building, University of Exeter, Stocker Road, Exeter, EX4 4QD, England

## Abstract

The asymmetric unit of the title salt, C_28_H_28_O_3_P^+^·Cl^−^·H_2_O, contains a benzyl­triphenyl­phospho­nium cation, a chloride counter-ion, and a water mol­ecule of crystallization. The 3,4,5-trimeth­oxy substituents of the benzylic functionality are arranged with the 3,5-methyl groups lying approximately in the aromatic ring plane while the 4-methyl group is out of the plane.

## Related literature

For background, see: Asakawa *et al.* (1976 ▶); Mervič *et al.* (1977 ▶); Lawrence *et al.* (2006 ▶).
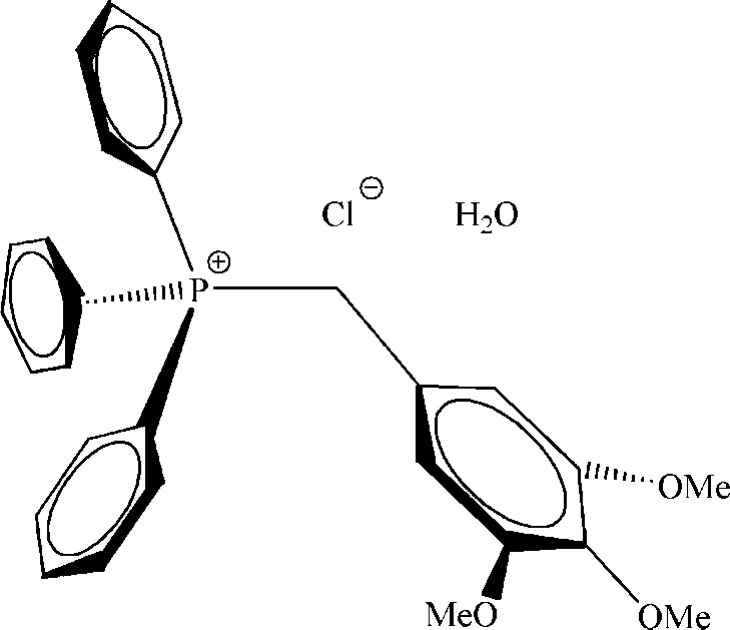


## Experimental

### 

#### Crystal data

C_28_H_28_O_3_P^+^·Cl^−^·H_2_O
*M* *_r_* = 496.94
Triclinic, 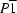

*a* = 10.5818 (8) Å
*b* = 10.6160 (15) Å
*c* = 13.8876 (15) Å
α = 111.020 (9)°
β = 95.895 (7)°
γ = 108.697 (11)°
*V* = 1337.0 (3) Å^3^
*Z* = 2
Mo *K*α radiation
μ = 0.23 mm^−1^
*T* = 294 (2) K
0.35 × 0.30 × 0.25 mm

#### Data collection

Bruker P4 diffractometer
Absorption correction: none
6874 measured reflections
5905 independent reflections
4821 reflections with *I* > 2σ(*I*)
*R*_int_ = 0.013
3 standard reflections every 147 reflections intensity decay: 0.5%

#### Refinement

*R*[*F* ^2^ > 2σ(*F* ^2^)] = 0.041
*wR*(*F* ^2^) = 0.112
*S* = 1.02
5905 reflections
313 parameters
H atoms treated by a mixture of independent and constrained refinement
Δρ_max_ = 0.31 e Å^−3^
Δρ_min_ = −0.33 e Å^−3^

### 

Data collection: *XSCANS* (Bruker, 1997 ▶); cell refinement: *XSCANS*; data reduction: *XSCANS*; program(s) used to solve structure: *SHELXS97* (Sheldrick, 2008 ▶); program(s) used to refine structure: *SHELXL97* (Sheldrick, 2008 ▶); molecular graphics: *SHELXTL* (Sheldrick, 2008 ▶); software used to prepare material for publication: *SHELXTL*.

## Supplementary Material

Crystal structure: contains datablocks I. DOI: 10.1107/S1600536808027712/fj2146sup1.cif

Structure factors: contains datablocks I. DOI: 10.1107/S1600536808027712/fj2146Isup2.hkl

Additional supplementary materials:  crystallographic information; 3D view; checkCIF report

## Figures and Tables

**Fig. 1.**
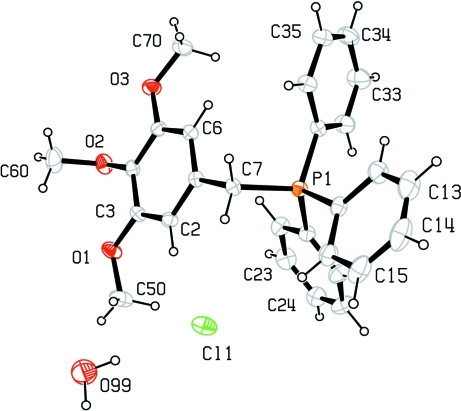
The molecular structure of the salt, with atom labels and 25% probability displacement ellipsoids for non-H atoms.

## supplementary crystallographic information

### Comment

The 3,4,5-trimethoxyphenyl group is found in a number of natural products with antifungal properties such as Brittonin A and B (Asakawa *et al.*, 1976), and central nervous system therapeutic properties such as Kadsurin (Mervič *et al.*, 1977). A number of anticancer chalcones also contain this functionality (Lawrence *et al.*, 2006); common synthetic routes to these products involve Wittig chemistry or Knoevenagel condensation reactions. The title compound is a Wittig precursor requiring deprotonation in the presence of a carbonyl compound.

The activity of the molecule classes above is believed to be related to the conformation of the 3,4,5-trimethoxyphenyl group relative to the other aromatic ring present. One aspect of this may be due to the disposition of the three methoxy groups, and the title molecule was chosen to provide a simple starting reference point for a more extensive study.

The structure obtained shows that O—C vectors are directed at 7, 81, and 8° to the phenyl ring plane for the 3,4, and 5-methoxy groups respectively.

### Experimental

The title compound was obtained from 3,4,5-trimethoxybenzyl alcohol in two steps.

The alcohol (20 g, 0.1 mol) was dissolved in diethylether (200 ml) and cooled to 0°C. Thionyl chloride (15 ml, 0.21 mol) was added dropwise over thirty minutes and the solution was stirred for two hours. Water (120 ml) was added portionwise and the ether layer was separated. Extraction of the aqueous layer with diethylether (3 *x* 25 ml), combination of the ether fractions, drying over granular calcium chloride, and removal of the solvent under reduced pressure gave white microcrystalline 3,4,5-trimethoxybenzyl chloride in near quantitative yield.

The product from the first stage was mixed with triphenylphosphine (37.2 g, 0.115 mol). Addition of toluene (200 ml) and pump-purging with nitrogen gave a colourless solution which was heated under nitrogen at reflux temperature for thirty hours. The reaction mixture was allowed to cool to room temperature before being filtered under nitrogen. The white microcrystalline product was washed with petroleum ether and dried under reduced pressure (42.1 g, 72%).

Crystallization of a small sample by layering petroleum ether (40–60°C) onto a concentrated dichloromethane solution produced crystals suitable for the structure determination.

### Refinement

H atoms bonded to the O atom were located in a difference map and refined with distance restraints of O—H = 0.84 Å, and with *U*_iso_(H) = 1.2*U*_eq_(O). Other H atoms were positioned geometrically and refined using a riding model, with C—H = 0.93–0.96 Å and with *U*_iso_(H) = 1.2 (1.5 for methyl groups) times *U*_eq_(C).

### Crystal data

**Table:**

C_28_H_28_O_3_P^+^·Cl^−^·H_2_O	*Z* = 2
*M**_r_* = 496.94	*F*(000) = 524
Triclinic, *P*1	*D*_x_ = 1.234 Mg m^−^^3^
Hall symbol: -P 1	Mo *K*α radiation, λ = 0.71073 Å
*a* = 10.5818 (8) Å	Cell parameters from 40 reflections
*b* = 10.6160 (15) Å	θ = 9.0–12.5°
*c* = 13.8876 (15) Å	µ = 0.23 mm^−^^1^
α = 111.020 (9)°	*T* = 294 K
β = 95.895 (7)°	Block, colourless
γ = 108.697 (11)°	0.35 × 0.30 × 0.25 mm
*V* = 1337.0 (3) Å^3^	

### Data collection

**Table:**

Bruker P4 diffractometer	*R*_int_ = 0.013
Radiation source: fine-focus sealed tube, Bruker P4	θ_max_ = 27.5°, θ_min_ = 1.8°
graphite	*h* = −13→1
ω scans	*k* = −12→12
6874 measured reflections	*l* = −18→18
5905 independent reflections	3 standard reflections every 147 reflections
4821 reflections with *I* > 2σ(*I*)	intensity decay: 0.5%

### Refinement

**Table:**

Refinement on *F*^2^	Primary atom site location: structure-invariant direct methods
Least-squares matrix: full	Secondary atom site location: difference Fourier map
*R*[*F*^2^ > 2σ(*F*^2^)] = 0.041	Hydrogen site location: inferred from neighbouring sites
*wR*(*F*^2^) = 0.112	H atoms treated by a mixture of independent and constrained refinement
*S* = 1.03	*w* = 1/[σ^2^(*F*_o_^2^) + (0.0485*P*)^2^ + 0.4603*P*] where *P* = (*F*_o_^2^ + 2*F*_c_^2^)/3
5905 reflections	(Δ/σ)_max_ < 0.001
313 parameters	Δρ_max_ = 0.31 e Å^−^^3^
0 restraints	Δρ_min_ = −0.33 e Å^−^^3^
0 constraints	

### Special details

**Table:**

Geometry. All e.s.d.'s (except the e.s.d. in the dihedral angle between two l.s. planes) are estimated using the full covariance matrix. The cell e.s.d.'s are taken into account individually in the estimation of e.s.d.'s in distances, angles and torsion angles; correlations between e.s.d.'s in cell parameters are only used when they are defined by crystal symmetry. An approximate (isotropic) treatment of cell e.s.d.'s is used for estimating e.s.d.'s involving l.s. planes.
Refinement. Refinement of *F*^2^ against ALL reflections. The weighted *R*-factor *wR* and goodness of fit *S* are based on *F*^2^, conventional *R*-factors *R* are based on *F*, with *F* set to zero for negative *F*^2^. The threshold expression of *F*^2^ > σ(*F*^2^) is used only for calculating *R*-factors(gt) *etc*. and is not relevant to the choice of reflections for refinement. *R*-factors based on *F*^2^ are statistically about twice as large as those based on *F*, and *R*- factors based on ALL data will be even larger.

### Fractional atomic coordinates and isotropic or equivalent isotropic displacement parameters (Å^2^)

**Table:**

	*x*	*y*	*z*	*U*_iso_*/*U*_eq_	
C1	0.59549 (16)	0.30260 (16)	0.18593 (12)	0.0346 (3)	
C2	0.73649 (16)	0.35116 (17)	0.19652 (12)	0.0369 (3)	
H2A	0.7780	0.2842	0.1741	0.044*	
C3	0.81545 (16)	0.49986 (17)	0.24067 (12)	0.0373 (3)	
C4	0.75394 (17)	0.60102 (16)	0.27451 (12)	0.0376 (3)	
C5	0.61208 (17)	0.55084 (17)	0.26445 (13)	0.0380 (3)	
C6	0.53270 (16)	0.40187 (17)	0.21970 (13)	0.0386 (3)	
H6A	0.4380	0.3688	0.2124	0.046*	
C7	0.51360 (16)	0.13991 (16)	0.13848 (12)	0.0366 (3)	
H7A	0.5683	0.0907	0.1009	0.055*	
H7B	0.4319	0.1174	0.0866	0.055*	
P1	0.46139 (4)	0.06666 (4)	0.23356 (3)	0.03399 (11)	
C12	0.2474 (2)	−0.2060 (2)	0.15299 (17)	0.0580 (5)	
H12A	0.1956	−0.1579	0.1890	0.070*	
C13	0.1887 (3)	−0.3548 (2)	0.0915 (2)	0.0779 (8)	
H13A	0.0966	−0.4065	0.0853	0.093*	
C14	0.2640 (3)	−0.4265 (2)	0.03979 (18)	0.0780 (8)	
H14A	0.2236	−0.5270	−0.0006	0.094*	
C15	0.4004 (3)	−0.3509 (2)	0.04689 (17)	0.0708 (7)	
H15A	0.4515	−0.4009	0.0116	0.085*	
C16	0.4617 (2)	−0.2005 (2)	0.10669 (15)	0.0525 (4)	
H16A	0.5529	−0.1490	0.1103	0.063*	
C11	0.38491 (18)	−0.12838 (17)	0.16085 (13)	0.0415 (4)	
C22	0.68223 (19)	0.26808 (19)	0.40368 (14)	0.0486 (4)	
H22A	0.6578	0.3386	0.3913	0.058*	
C23	0.7940 (2)	0.3093 (2)	0.48553 (16)	0.0608 (5)	
H23A	0.8445	0.4081	0.5279	0.073*	
C24	0.8313 (2)	0.2062 (3)	0.50507 (17)	0.0655 (6)	
H24A	0.9064	0.2351	0.5604	0.079*	
C25	0.7571 (2)	0.0608 (3)	0.44254 (18)	0.0672 (6)	
H25A	0.7821	−0.0090	0.4556	0.081*	
C26	0.6446 (2)	0.0167 (2)	0.35967 (16)	0.0529 (4)	
H26A	0.5950	−0.0824	0.3175	0.064*	
C21	0.60641 (16)	0.11974 (18)	0.33980 (13)	0.0384 (3)	
C32	0.3431 (2)	0.1799 (2)	0.39684 (15)	0.0560 (5)	
H32A	0.4150	0.1823	0.4429	0.067*	
C33	0.2424 (3)	0.2246 (3)	0.43595 (17)	0.0705 (6)	
H33A	0.2467	0.2570	0.5084	0.085*	
C34	0.1362 (2)	0.2213 (3)	0.36822 (18)	0.0630 (5)	
H34A	0.0696	0.2528	0.3952	0.076*	
C35	0.12752 (19)	0.1718 (2)	0.26053 (16)	0.0537 (5)	
H35A	0.0544	0.1682	0.2149	0.064*	
C36	0.22765 (18)	0.12755 (19)	0.22064 (14)	0.0449 (4)	
H36A	0.2225	0.0950	0.1481	0.054*	
C31	0.33660 (16)	0.13157 (17)	0.28894 (13)	0.0381 (3)	
O1	0.95480 (12)	0.55746 (13)	0.25460 (11)	0.0512 (3)	
O2	0.83251 (14)	0.74874 (12)	0.32194 (10)	0.0508 (3)	
O3	0.56099 (14)	0.65759 (14)	0.30251 (12)	0.0531 (3)	
C50	1.0203 (2)	0.4558 (2)	0.2316 (2)	0.0654 (6)	
H50A	1.1176	0.5071	0.2444	0.098*	
H50B	0.9839	0.3872	0.1583	0.098*	
H50C	1.0036	0.4044	0.2764	0.098*	
C60	0.8700 (3)	0.8112 (2)	0.2485 (2)	0.0795 (8)	
H60A	0.9240	0.9140	0.2869	0.119*	
H60B	0.7883	0.7968	0.2017	0.119*	
H60C	0.9229	0.7648	0.2075	0.119*	
C70	0.4212 (2)	0.6117 (2)	0.3081 (2)	0.0636 (6)	
H70A	0.3970	0.6955	0.3349	0.095*	
H70B	0.4090	0.5630	0.3548	0.095*	
H70C	0.3631	0.5458	0.2383	0.095*	
Cl1	0.79294 (5)	0.01474 (6)	0.06989 (4)	0.05847 (15)	
O99	1.0516 (3)	0.2095 (2)	−0.0017 (2)	0.1006 (7)	
H99A	1.095 (5)	0.153 (5)	−0.021 (3)	0.151*	
H99B	0.982 (5)	0.154 (5)	0.016 (3)	0.151*	

### Atomic displacement parameters (Å^2^)

**Table:**

	*U*^11^	*U*^22^	*U*^33^	*U*^12^	*U*^13^	*U*^23^
C1	0.0353 (8)	0.0333 (7)	0.0341 (7)	0.0111 (6)	0.0093 (6)	0.0143 (6)
C2	0.0343 (8)	0.0346 (8)	0.0399 (8)	0.0135 (6)	0.0105 (6)	0.0126 (6)
C3	0.0311 (7)	0.0384 (8)	0.0379 (8)	0.0099 (6)	0.0087 (6)	0.0138 (6)
C4	0.0400 (8)	0.0300 (7)	0.0375 (8)	0.0090 (6)	0.0110 (6)	0.0116 (6)
C5	0.0409 (8)	0.0366 (8)	0.0415 (8)	0.0183 (7)	0.0133 (7)	0.0180 (7)
C6	0.0329 (8)	0.0390 (8)	0.0449 (9)	0.0132 (6)	0.0105 (6)	0.0187 (7)
C7	0.0346 (8)	0.0342 (8)	0.0361 (8)	0.0100 (6)	0.0093 (6)	0.0118 (6)
P1	0.03064 (19)	0.03014 (19)	0.0356 (2)	0.00880 (15)	0.00623 (15)	0.01065 (15)
C12	0.0451 (10)	0.0435 (10)	0.0684 (13)	0.0021 (8)	0.0025 (9)	0.0208 (9)
C13	0.0682 (15)	0.0455 (12)	0.0816 (16)	−0.0120 (11)	−0.0115 (13)	0.0226 (12)
C14	0.112 (2)	0.0323 (10)	0.0544 (12)	0.0001 (12)	−0.0074 (13)	0.0113 (9)
C15	0.122 (2)	0.0448 (11)	0.0487 (11)	0.0387 (13)	0.0235 (12)	0.0161 (9)
C16	0.0687 (12)	0.0404 (9)	0.0480 (10)	0.0202 (9)	0.0172 (9)	0.0178 (8)
C11	0.0442 (9)	0.0326 (8)	0.0392 (8)	0.0080 (7)	0.0036 (7)	0.0133 (6)
C22	0.0499 (10)	0.0407 (9)	0.0444 (9)	0.0121 (8)	0.0030 (8)	0.0130 (7)
C23	0.0533 (11)	0.0555 (11)	0.0454 (10)	0.0058 (9)	−0.0045 (9)	0.0080 (9)
C24	0.0510 (11)	0.0850 (16)	0.0502 (11)	0.0270 (11)	−0.0031 (9)	0.0202 (11)
C25	0.0725 (14)	0.0727 (14)	0.0609 (13)	0.0404 (12)	0.0008 (11)	0.0259 (11)
C26	0.0563 (11)	0.0456 (10)	0.0520 (10)	0.0211 (9)	0.0019 (8)	0.0168 (8)
C21	0.0344 (8)	0.0380 (8)	0.0376 (8)	0.0118 (6)	0.0061 (6)	0.0128 (6)
C32	0.0555 (11)	0.0788 (14)	0.0410 (9)	0.0340 (10)	0.0164 (8)	0.0246 (9)
C33	0.0757 (15)	0.0980 (18)	0.0470 (11)	0.0454 (14)	0.0307 (11)	0.0256 (11)
C34	0.0559 (12)	0.0736 (14)	0.0702 (13)	0.0359 (11)	0.0319 (11)	0.0273 (11)
C35	0.0397 (9)	0.0608 (11)	0.0604 (11)	0.0224 (9)	0.0116 (8)	0.0226 (9)
C36	0.0405 (9)	0.0494 (10)	0.0404 (9)	0.0172 (8)	0.0083 (7)	0.0143 (7)
C31	0.0352 (8)	0.0386 (8)	0.0375 (8)	0.0122 (6)	0.0113 (6)	0.0139 (6)
O1	0.0307 (6)	0.0434 (7)	0.0654 (8)	0.0083 (5)	0.0126 (5)	0.0122 (6)
O2	0.0550 (7)	0.0310 (6)	0.0530 (7)	0.0068 (5)	0.0177 (6)	0.0102 (5)
O3	0.0512 (7)	0.0418 (7)	0.0751 (9)	0.0260 (6)	0.0240 (7)	0.0242 (6)
C50	0.0370 (10)	0.0626 (13)	0.0881 (16)	0.0218 (9)	0.0163 (10)	0.0193 (11)
C60	0.104 (2)	0.0470 (12)	0.0939 (18)	0.0200 (12)	0.0490 (16)	0.0374 (12)
C70	0.0555 (12)	0.0657 (13)	0.0885 (16)	0.0382 (10)	0.0347 (11)	0.0349 (12)
Cl1	0.0422 (2)	0.0691 (3)	0.0547 (3)	0.0258 (2)	0.00903 (19)	0.0125 (2)
O99	0.1220 (19)	0.0785 (13)	0.1320 (18)	0.0569 (13)	0.0683 (15)	0.0509 (13)

### Geometric parameters (Å, °)

**Table:**

C1—C2	1.386 (2)	C23—H23A	0.9300
C1—C6	1.389 (2)	C24—C25	1.369 (3)
C1—C7	1.508 (2)	C24—H24A	0.9300
C2—C3	1.386 (2)	C25—C26	1.391 (3)
C2—H2A	0.9300	C25—H25A	0.9300
C3—O1	1.3654 (19)	C26—C21	1.384 (3)
C3—C4	1.395 (2)	C26—H26A	0.9300
C4—O2	1.3766 (19)	C32—C33	1.384 (3)
C4—C5	1.396 (2)	C32—C31	1.385 (2)
C5—O3	1.3704 (19)	C32—H32A	0.9300
C5—C6	1.389 (2)	C33—C34	1.372 (3)
C6—H6A	0.9300	C33—H33A	0.9300
C7—P1	1.8069 (16)	C34—C35	1.378 (3)
C7—H7A	0.9700	C34—H34A	0.9300
C7—H7B	0.9700	C35—C36	1.379 (3)
P1—C11	1.7943 (17)	C35—H35A	0.9300
P1—C31	1.7949 (17)	C36—C31	1.395 (2)
P1—C21	1.7982 (16)	C36—H36A	0.9300
C12—C13	1.380 (3)	O1—C50	1.423 (2)
C12—C11	1.393 (3)	O2—C60	1.427 (3)
C12—H12A	0.9300	O3—C70	1.421 (2)
C13—C14	1.358 (4)	C50—H50A	0.9600
C13—H13A	0.9300	C50—H50B	0.9600
C14—C15	1.381 (4)	C50—H50C	0.9600
C14—H14A	0.9300	C60—H60A	0.9600
C15—C16	1.391 (3)	C60—H60B	0.9600
C15—H15A	0.9300	C60—H60C	0.9600
C16—C11	1.387 (3)	C70—H70A	0.9600
C16—H16A	0.9300	C70—H70B	0.9600
C22—C23	1.385 (3)	C70—H70C	0.9600
C22—C21	1.397 (2)	O99—H99A	0.85 (4)
C22—H22A	0.9300	O99—H99B	0.89 (4)
C23—C24	1.377 (3)		
			
C2—C1—C6	120.50 (14)	C22—C23—H23A	119.5
C2—C1—C7	118.08 (14)	C25—C24—C23	119.54 (19)
C6—C1—C7	121.41 (14)	C25—C24—H24A	120.2
C3—C2—C1	119.79 (15)	C23—C24—H24A	120.2
C3—C2—H2A	120.1	C24—C25—C26	120.6 (2)
C1—C2—H2A	120.1	C24—C25—H25A	119.7
O1—C3—C2	123.80 (15)	C26—C25—H25A	119.7
O1—C3—C4	115.76 (14)	C21—C26—C25	120.13 (18)
C2—C3—C4	120.44 (14)	C21—C26—H26A	119.9
O2—C4—C3	120.66 (15)	C25—C26—H26A	119.9
O2—C4—C5	120.02 (15)	C26—C21—C22	119.22 (16)
C3—C4—C5	119.26 (14)	C26—C21—P1	121.36 (13)
O3—C5—C6	124.28 (15)	C22—C21—P1	119.41 (13)
O3—C5—C4	115.34 (14)	C33—C32—C31	119.84 (19)
C6—C5—C4	120.37 (15)	C33—C32—H32A	120.1
C5—C6—C1	119.63 (15)	C31—C32—H32A	120.1
C5—C6—H6A	120.2	C34—C33—C32	120.24 (19)
C1—C6—H6A	120.2	C34—C33—H33A	119.9
C1—C7—P1	114.83 (11)	C32—C33—H33A	119.9
C1—C7—H7A	108.6	C33—C34—C35	120.53 (19)
P1—C7—H7A	108.6	C33—C34—H34A	119.7
C1—C7—H7B	108.6	C35—C34—H34A	119.7
P1—C7—H7B	108.6	C34—C35—C36	119.77 (18)
H7A—C7—H7B	107.5	C34—C35—H35A	120.1
C11—P1—C31	109.68 (8)	C36—C35—H35A	120.1
C11—P1—C21	110.92 (8)	C35—C36—C31	120.13 (16)
C31—P1—C21	108.89 (8)	C35—C36—H36A	119.9
C11—P1—C7	106.24 (7)	C31—C36—H36A	119.9
C31—P1—C7	110.07 (8)	C32—C31—C36	119.49 (16)
C21—P1—C7	111.01 (8)	C32—C31—P1	121.59 (14)
C13—C12—C11	119.6 (2)	C36—C31—P1	118.87 (12)
C13—C12—H12A	120.2	C3—O1—C50	116.07 (14)
C11—C12—H12A	120.2	C4—O2—C60	114.00 (15)
C14—C13—C12	120.6 (2)	C5—O3—C70	117.11 (14)
C14—C13—H13A	119.7	O1—C50—H50A	109.5
C12—C13—H13A	119.7	O1—C50—H50B	109.5
C13—C14—C15	120.4 (2)	H50A—C50—H50B	109.5
C13—C14—H14A	119.8	O1—C50—H50C	109.5
C15—C14—H14A	119.8	H50A—C50—H50C	109.5
C14—C15—C16	120.3 (2)	H50B—C50—H50C	109.5
C14—C15—H15A	119.9	O2—C60—H60A	109.5
C16—C15—H15A	119.9	O2—C60—H60B	109.5
C11—C16—C15	119.1 (2)	H60A—C60—H60B	109.5
C11—C16—H16A	120.5	O2—C60—H60C	109.5
C15—C16—H16A	120.5	H60A—C60—H60C	109.5
C16—C11—C12	120.02 (17)	H60B—C60—H60C	109.5
C16—C11—P1	119.19 (14)	O3—C70—H70A	109.5
C12—C11—P1	120.66 (15)	O3—C70—H70B	109.5
C23—C22—C21	119.55 (18)	H70A—C70—H70B	109.5
C23—C22—H22A	120.2	O3—C70—H70C	109.5
C21—C22—H22A	120.2	H70A—C70—H70C	109.5
C24—C23—C22	120.96 (19)	H70B—C70—H70C	109.5
C24—C23—H23A	119.5	H99A—O99—H99B	100 (4)
			
C6—C1—C2—C3	−0.3 (2)	C21—C22—C23—C24	−0.1 (3)
C7—C1—C2—C3	−179.41 (14)	C22—C23—C24—C25	0.1 (4)
C1—C2—C3—O1	179.69 (15)	C23—C24—C25—C26	0.0 (4)
C1—C2—C3—C4	0.0 (2)	C24—C25—C26—C21	−0.2 (4)
O1—C3—C4—O2	−2.1 (2)	C25—C26—C21—C22	0.2 (3)
C2—C3—C4—O2	177.63 (14)	C25—C26—C21—P1	−179.85 (17)
O1—C3—C4—C5	−179.06 (15)	C23—C22—C21—C26	0.0 (3)
C2—C3—C4—C5	0.7 (2)	C23—C22—C21—P1	−179.99 (16)
O2—C4—C5—O3	1.2 (2)	C11—P1—C21—C26	−1.85 (18)
C3—C4—C5—O3	178.17 (14)	C31—P1—C21—C26	118.94 (16)
O2—C4—C5—C6	−178.00 (14)	C7—P1—C21—C26	−119.73 (16)
C3—C4—C5—C6	−1.0 (2)	C11—P1—C21—C22	178.12 (14)
O3—C5—C6—C1	−178.40 (15)	C31—P1—C21—C22	−61.09 (16)
C4—C5—C6—C1	0.7 (2)	C7—P1—C21—C22	60.24 (16)
C2—C1—C6—C5	0.0 (2)	C31—C32—C33—C34	0.0 (4)
C7—C1—C6—C5	179.03 (14)	C32—C33—C34—C35	0.9 (4)
C2—C1—C7—P1	103.56 (15)	C33—C34—C35—C36	−1.2 (3)
C6—C1—C7—P1	−75.54 (17)	C34—C35—C36—C31	0.6 (3)
C1—C7—P1—C11	−173.31 (12)	C33—C32—C31—C36	−0.5 (3)
C1—C7—P1—C31	68.01 (13)	C33—C32—C31—P1	−177.89 (18)
C1—C7—P1—C21	−52.62 (14)	C35—C36—C31—C32	0.2 (3)
C11—C12—C13—C14	0.9 (4)	C35—C36—C31—P1	177.66 (14)
C12—C13—C14—C15	−0.9 (4)	C11—P1—C31—C32	107.02 (17)
C13—C14—C15—C16	−0.3 (4)	C21—P1—C31—C32	−14.53 (18)
C14—C15—C16—C11	1.5 (3)	C7—P1—C31—C32	−136.43 (16)
C15—C16—C11—C12	−1.4 (3)	C11—P1—C31—C36	−70.42 (15)
C15—C16—C11—P1	−177.44 (15)	C21—P1—C31—C36	168.03 (13)
C13—C12—C11—C16	0.3 (3)	C7—P1—C31—C36	46.13 (15)
C13—C12—C11—P1	176.19 (17)	C2—C3—O1—C50	−6.6 (3)
C31—P1—C11—C16	176.66 (14)	C4—C3—O1—C50	173.14 (17)
C21—P1—C11—C16	−63.01 (16)	C3—C4—O2—C60	81.1 (2)
C7—P1—C11—C16	57.73 (16)	C5—C4—O2—C60	−102.0 (2)
C31—P1—C11—C12	0.69 (18)	C6—C5—O3—C70	8.1 (3)
C21—P1—C11—C12	121.01 (16)	C4—C5—O3—C70	−171.10 (17)
C7—P1—C11—C12	−118.25 (16)		
